# Inhibition of Hypoxia-Induced Retinal Angiogenesis by Specnuezhenide, an Effective Constituent of *Ligustrum lucidum* Ait., through Suppression of the HIF-1α/VEGF Signaling Pathway

**DOI:** 10.3390/molecules21121756

**Published:** 2016-12-21

**Authors:** Jianming Wu, Xiao Ke, Wei Fu, Xiaoping Gao, Hongcheng Zhang, Wei Wang, Na Ma, Manxi Zhao, Xiaofeng Hao, Zhirong Zhang

**Affiliations:** 1 Laboratory of Chinese Materia Medica, Department of Pharmacology, School of Pharmacy, Southwest Medical University, Luzhou 646000, Sichuan, China; jianmingwu@swmu.edu.cn; 2 Post-Doctoral Research Station, KangHong Pharmaceutical Group, Chengdu 610036, Sichuan, China; kexiao@cnkh.com (X.K.); fu_wei99@163.com (W.F.); 1483687108@qq.com (X.G.); arken20@163.com (H.Z.); dy_ts110@163.com (W.W.); manapppp@163.com (N.M.); zhaomanqian@cnkh.com (M.Z.); 3 Post-Doctoral Mobile Station, West China School of Pharmacy, Sichuan University, Chengdu 610041, Sichuan, China

**Keywords:** specnuezhenide, angiogenesis, hypoxia-inducible factor-1, oxygen-induced retinopathy, vascular endothelial growth factor

## Abstract

Specnuezhenide (SPN), one of the main ingredients of Chinese medicine “Nü-zhen-zi”, has anti-angiogenic and vision improvement effects. However, studies of its effect on retinal neovascularization are limited so far. In the present study, we established a vascular endothelial growth factor A (VEGFA) secretion model of human acute retinal pigment epithelial-19 (ARPE-19) cells by exposure of 150 μM CoCl_2_ to the cells and determined the VEGFA concentrations, the mRNA expressions of VEGFA, hypoxia inducible factor-1α (HIF-1α) & prolyl hydroxylases 2 (PHD-2), and the protein expressions of HIF-1α and PHD-2 after treatment of 3-(5′-hydroxymethyl-2′-furyl)-1-benzylindazole (YC-1, 1.0 μg/mL) or SPN (0.2, 1.0 and 5.0 μg/mL). Furthermore, rat pups with retinopathy were treated with SPN (5.0 and 10.0 mg/kg) in an 80% oxygen atmosphere and the retinal avascular areas were assessed through visualization using infusion of ADPase and H&E stains. The results showed that SPN inhibited VEGFA secretion by ARPE-19 cells under hypoxia condition, down-regulated the mRNA expressions of VEGFA and PHD-2 slightly, and the protein expressions of VEGFA, HIF-1α and PHD-2 significantly in vitro. SPN also prevented hypoxia-induced retinal neovascularization in a rat model of oxygen-induced retinopathy in vivo. These results indicate that SPN ameliorates retinal neovascularization through inhibition of HIF-1α/VEGF signaling pathway. Therefore, SPN has the potential to be developed as an agent for the prevention and treatment of diabetic retinopathy.

## 1. Introduction

**Figure 1:**
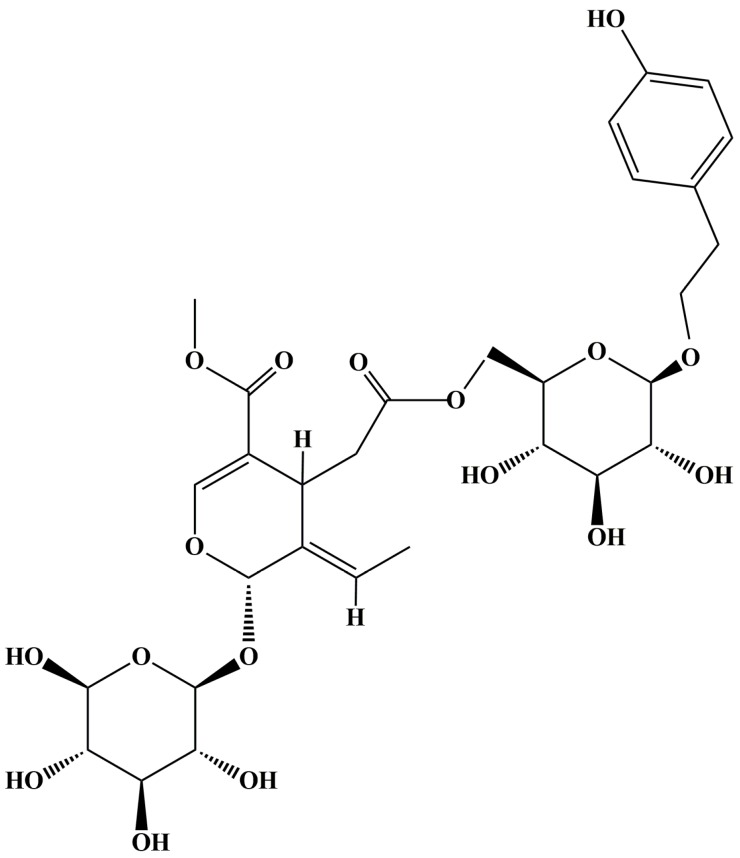
Structure of methyl (5*E*,6*S*)-5-ethylidene-4-[2-oxo-2-[[(2*R*,3*S*,4*S*,5*R*,6*R*)-3,4,5-trihydroxy-6-[2-(4-hydroxyphenyl)ethoxy]oxan-2-yl]methoxy]ethyl]-6-[(2*S*,3*R*,4*S*,5*S*,6*R*)-3,4,5-trihydroxy-6-(hydroxymethyl)oxan-2-yl]oxy-4*H*-pyran-3-carboxylate (specnuezhenide, SPN).

The fruit of *Ligustrum lucidum* Ait. (*L. lucidum*), known as “Nü-zhen-zi” in Chinese, is a famous traditional Chinese medicine with liver-, kidney- and vision-improving functions [1,2]. As a well-known Chinese traditional medicine, the use of *L. lucidum* for its immunomodulation [3], antioxidant [4,5,6], anti-cancer [7], hypolipidemia [8], neuroprotection [9], hypoglycemia [10,11], anti-atherosclerosis [12], periodontal pathogen inhibition [13], hepatoprotection [14], antiviral [15], anti-osteoporosis [16], anti-obesity [17], anti-hepatitis C virus [18] and other activities has been reported. Meanwhile, phytochemical studies have revealed the main chemical components of *L. lucidum* to include terpenoids, iridoid glycosides, flavonoids, phenethyl alcohol glycosides, volatile oils, phospholipids, polysaccharides, fatty acids, amino acids and so on [19]. Among them, methyl (5*E*,6*S*)-5-ethylidene-4-[2-oxo-2-[[(2*R*,3*S*,4*S*,5*R*,6*R*)-3,4,5-trihydroxy-6-[2-(4-hydroxyphenyl)ethoxy]oxan-2-yl]methoxy]ethyl]-6-[(2*S*,3*R*,4*S*,5*S*,6*R*)-3,4,5-trihydroxy-6-(hydroxylmethyl)oxan-2-yl]oxy-4*H*-pyran-3-carboxylate (specnuezhenide, SPN, Figure 1), an iridoid glycoside with immune-strengthening functions in murine splenocytes and anti-angiogenic effects in human umbilical vein endothelial cells [20,21], has been frequently used as the quality control marker for *L. lucidum* and its preparations. Recently, it has been reported that some preparations mainly composed of *L. lucidum*, such as Tangmuning decoction and Keluoxin capsule, could alleviate the retinal neovascularization of diabetic retinopathy (DR) [22,23]. Although SPN exhibited antiangiogenic effects on human umbilical vein endothelial cells [21], it is still unknown whether SPN can inhibit hypoxia-induced retinal neovascularization in the pathophysiologic process of DR. 

DR is a common microvascular complication in the patients with diabetes mellitus [24]. Retinal neovascularization may result in vitreous hemorrhage and tractional retinal detachment [25]. Furthermore, increased vascular permeability may lead to macular edema in patients with DR [26], making DR a leading cause of blindness among the working population. Vascular endothelial growth factor A (VEGFA) which stimulates the proliferation and migration of vascular endothelial cells and increases vascular permeability, plays a critical role in the retinal neovascularization of DR [27]. Meanwhile, retinal pigment epithelium (RPE) cells located between the photoreceptors and choriocapillaris (capillaries forming the inner vascular layer of the choroid) play an important role in maintaining retinal homeostasis and VEGF production within the area of blood–ocular barrier [28,29]. Therefore, human acute retinal pigment epithelial-19 (ARPE-19) cells were usually used as an important cell model for study of VEGF secretion in vitro.

Hypoxia is one of the most potent triggers of VEGFA expression during the processes of DNA transcription, mRNA stabilization, translation and release of VEGFA [30]. Hypoxia is centrally controlled by hypoxia inducible factor-1α (HIF-1α), a transcription factor for regulation of hypoxia-inducible genes such as VEGFA for angiogenic response [31]. Therefore, HIF-1α is increased for induction of the expression of VEGFA under hypoxia, resulting in increased vascular permeability and retinal neovascularization. On the other hand, inhibition of HIF-1α can prevent the retinal neovascularization in the condition of hypoxia [32,33,34]. HIF-1α can be hydroxylated by prolyl hydroxylases (PHDs), especially PHD-2 in normoxia. The hydroxylated HIF-1α is multiubiquitinated and degraded in the proteasome. Conversely, the mRNA expression of PHD-2 is upregulated by hypoxia through an HIF-1α-dependent signaling pathway. In addition, the PHD activity is inhibited under low oxygen tension [35,36]. However, CoCl_2_ can inhibit the activity of PHD-2 to cause the accumulation of HIF and overexpression of VEGFA. Those revelations suggest that HIF-1α/VEGF signaling pathway plays the key role in the retinal neovascularization of DR. 

Oxygen-induced retinopathy (OIR) is the most important model of DR. The rat retina is highly immature at birth and the retinal vessels arise from mesenchymal precursors like those of humans, but contrary to humans, canalization of the inner retinal vessels of rat is not related to the presence of cystoid spaces. This extreme immaturity makes the rat retina highly susceptible to direct damage from oxygen. OIR can be produced by exposing newborn rat to 80% oxygen during the first 7–10 days of life [37]. In the present study, we investigated the preventive effect of SPN on retinal neovascularization from secretion of VEGFA in ARPE-19 cells induced by CoCl_2_ in vitro and neovascularization of OIR of rat model in vivo.

## 2. Results

### 2.1. SPN Inhibits the Secretion of VEGFA in ARPE-19 Cells under Hypoxia

**Figure 2:**
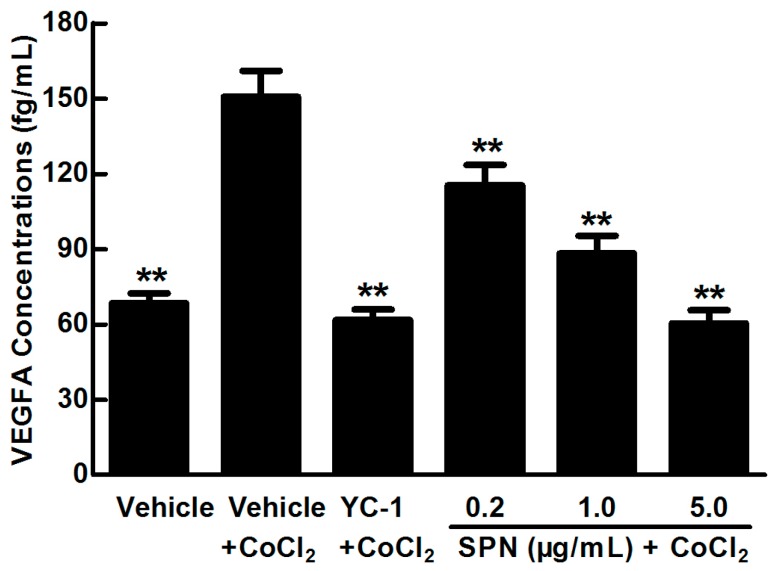
Effect of SPN on VEGFA secretion in ARPE-19 cells at 48 h of hypoxic condition. The results are representative of at least three independent experiments run in triplicate and expressed as the mean ± SD (*n* = 9). ** *p* < 0.01 vs. vehicle plus CoCl_2_-treated group. The control cells were treated with vehicle (culture medium containing 0.1% DMSO).

SPN is an important compound in *L. lucidum* for the vision protection of rats with DR. To study the effect of SPN on VEGFA secretion under hypoxia, enzyme-linked immunosorbent assay (ELISA) was applied to examine the concentrations of VEGFA in the cultured medium of ARPE-19 cells treated with 150 µM CoCl_2_ alone or in combination with SPN at 0.2, 1.0 or 5.0 μg/mL and compared to positive control of YC-1. As shown in Figure 2, the level of VEGFA in culture supernatant is significantly increased after treatment with 150 μM CoCl_2_ compared to that of the control (*p* < 0.01). However, YC-1 at 1.0 μg/mL and SPN at 0.2, 1.0 and 5.0 μg/mL markedly decreased the secretion of VEGFA compared to that of the cells treated with CoCl_2_ and vehicle (*p* < 0.01). These results indicate that SPN inhibits the secretion of VEGFA in the culture supernatants of ARPE-19 cells.

### 2.2. Effects of SPN on the mRNA Expressions of VEGFA, HIF-1α and PHD-2 in ARPE-19 Cells

**Figure 3:**
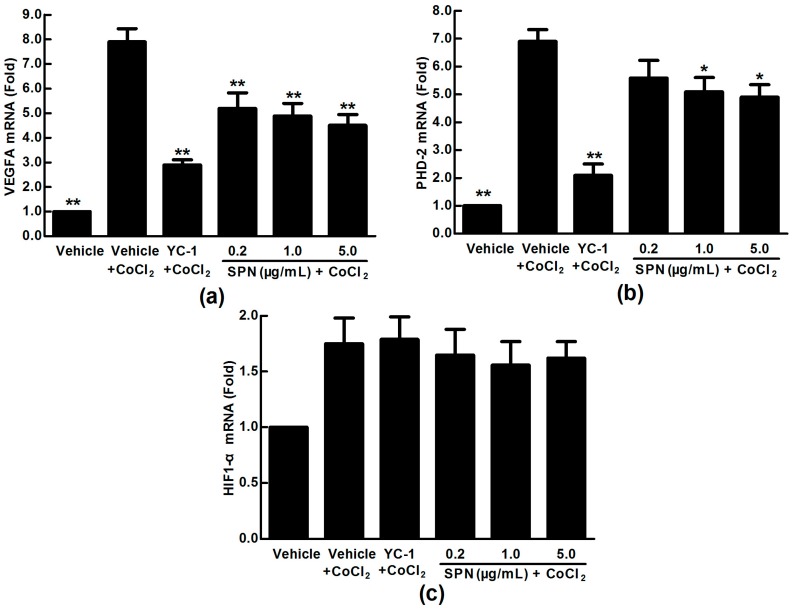
Effects of SPN on the mRNA expressions of VEGFA, HIF-1α and PHD-2 in ARPE-19 cells at 24 h of normoxia or hypoxic conditions. (**a**): VEGFA mRNA expression; (**b**): HIF-1α mRNA expression; (**c**): PHD-2 mRNA expression. The results are representative of at least three independent experiments run in triplicate and expressed as the mean ± SD (*n* = 9). * *p* < 0.05, ** *p* < 0.01 vs. vehicle plus CoCl_2_-treated group. The control cells were treated with vehicle (culture medium containing 0.1% DMSO).

VEGFA expression in pathophysiological states can be induced by post-transcriptional mechanisms directed at the VEGFA mRNA or its relative regulator [38]. We hypothesized that SPN may be involved in mRNA expressions of VEGFA, HIF-1α and PHD-2. For this purpose, we used qRT-PCR analysis to examine the mRNA expressions of VEGFA, HIF-1α and PHD-2 in the ARPE-19 cells. As shown in Figure 3, the expression of VEGFA mRNA was increased significantly in ARPE-19 cells treated with 150 μM CoCl_2_ for 24 h (*p* < 0.01) (Figure 3a). Adding SPN at 0.2–5.0 μg/mL significantly decreased the mRNA expression of VEGFA (*p* < 0.05 or *p* < 0.01) at a dose-dependent manner (Figure 3a), indicating that SPN blocked the mRNA expression of VEGFA induced by hypoxia. The data in Figure 3b show that 150 μM CoCl_2_, YC-1 at 1.0 μg/mL or SPN at 0.2–5.0 μg/mL do not affect the mRNA expression of HIF-1α significantly. However, the data in Figure 3c show that the mRNA expression of PHD-2 was increased (*p* < 0.01) under CoCl_2_ hypoxia-mimicking conditions, while it is significantly down-regulated in CoCl_2_ plus YC-1- or CoCl_2_ plus SPN-treated groups (1.0 μg/mL and 5.0 μg/mL) compared to that of CoCl_2_-treated group (*p* < 0.05 or *p* < 0.01). These results indicate that VEGFA protein secretion under hypoxia may be associated with the mRNA expression of VEGFA and PHD-2.

### 2.3. Effects of SPN on the Intracellular Protein Levels of HIF-1α and PHD-2 in ARPE-19 Cells

**Figure 4:**
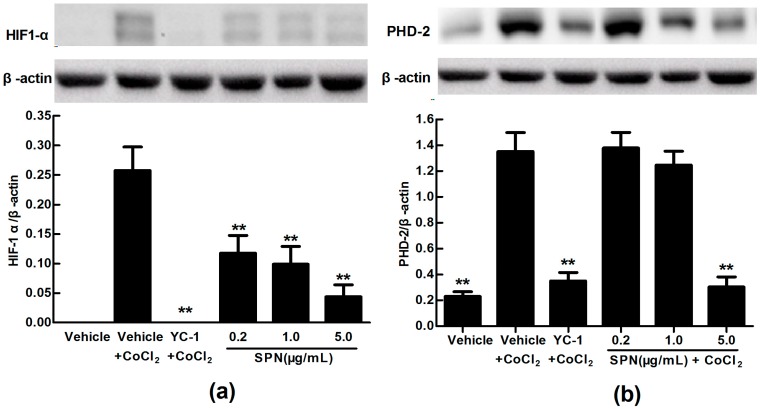
Effects of SPN on the protein expressions of HIF-1α and PHD-2 in ARPE-19 cells at 24 h of normoxia or hypoxic conditions. (**a**): HIF-1α protein expression; (**b**): PHD-2 protein expression. The results are representative of at least three independent experiments run in triplicate and expressed as the mean ± SD (*n* = 9). ** *p* < 0.01 vs. vehicle plus CoCl_2_-treated group. The control cells were treated with vehicle (culture medium containing 0.1% DMSO). Beta-actin was used as the loading control.

VEGFA is potently induced by hypoxia and regulated by HIF-1α and its degradation regulator PHD-2 [39]. Here, we used anti-HIF-1α and anti-PHD-2 antibodies to examine the expressions of HIF-1α and PHD-2 in cultured ARPE-19 cells under hypoxia condition. Western blot analysis showed that the protein expression levels of HIF-1α and PHD-2 were up-regulated significantly in ARPE-19 cells treated with 150 µM CoCl_2_ for 24 h compared to that of control treated with medium (*p* < 0.01). However, the protein expression level of HIF-1α was significantly down-regulated in CoCl_2_ plus YC-1- or CoCl_2_ plus SPN-treated groups (0.2, 1.0 and 5.0 μg/mL) compared to that of vehicle plus CoCl_2_-treated group (*p* < 0.01) (Figure 4a). Meanwhile, the protein expression level of PHD-2 was decreased in CoCl_2_ plus YC-1- or CoCl_2_ plus SPN-treated groups (5.0 μg/mL) compared to that of vehicle plus CoCl_2_-treated group (*p* < 0.01) because of HIF-1α acute reduction (Figure 4b).

These results indicate that the inhibition of SPN on VEGFA secretion of ARPE-19 cells under hypoxia is associated with the protein expressions of HIF-1α and PHD-2, suggesting a possible mechanism involved in hypoxia-induced retinal neovascularization and related signaling pathways.

### 2.4. SPN Inhibits Hypoxia-Induced Retinal Neovascularization In Vivo

**Figure 5:**
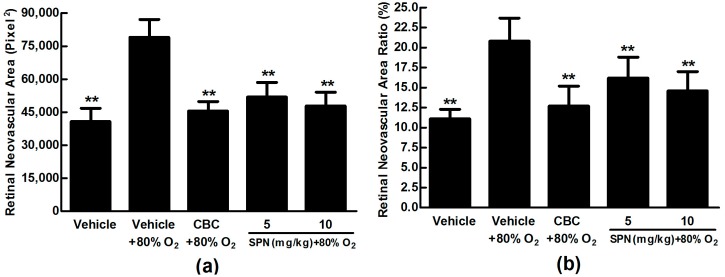
Effects of SPN and Conbercept (CBC) on quantification of retinal neovascular area ration in the rat model of oxygen-induced retinopathy (OIR). (**a**): Retinal neovascular area ration. (**b**): Inhibition of retinal neovascular area. The results were expressed as means ± SD (*n* = 10). ** *p* < 0.01, compared with vehicle plus 80% oxygen group.

**Figure 6:**
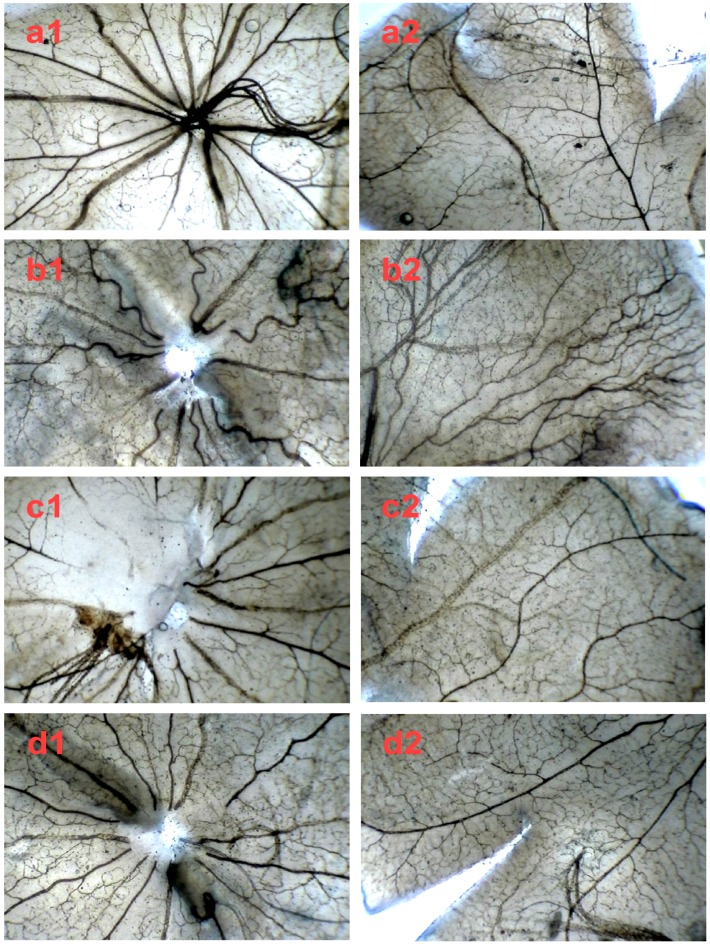

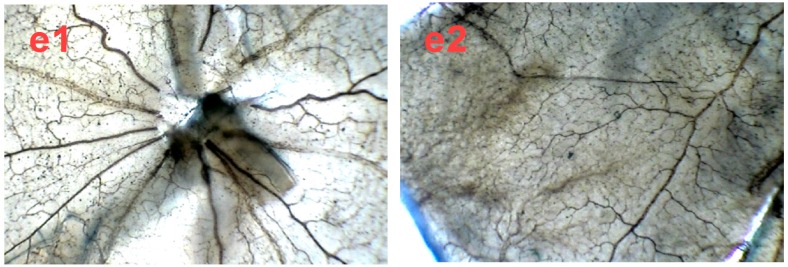
The in vivo effect of SPN and Conbercept on retinopathy of rats in ADPase-stained retinal sections (40×). Central retinal region of rat pups fostered under normoxia conditions (**a1**); OIR rats intraperitoneally administered with NS (**b1**), 1.0 mg/kg Conbercept (**c1**), 5.0 mg/kg SPN (**d1**) or 10.0 mg/kg SPN (**e1**). Peripheral retinal region of control, rat pups fostered under normoxia conditions (**a2**); OIR rats intraperitoneally administered with NS (**b2**), 1.0 mg/kg Conbercept (**c2**), 5.0 mg/kg SPN (**d2**) or 10.0 mg/kg SPN (**e2**). Ten rats were used for each group (*n* = 10).

After we demonstrated the inhibitory effect of SPN on VEGFA secretion in ARPE-19 cells in vitro, next, we further investigated the preventive effect of SPN on the hypoxia-induced retinal neovascularization with superficial vascular plexuses using ADPase staining in a rat pup model. The rat pups were exposed to 80% oxygen from postnatal day (PD) 7 to PD 12 to produce retinal and then returned to room air. This condition made the retina relatively hypoxic, resulting in the formation of retinal neovascularization. On PD 17, the OIR rats treated with vehicle increased the total retinal neovascular areas significantly compared to that of the normal rats treated with the vehicle (*p* < 0.01), whereas SPN markedly reversed the hypoxia-induced total neovascular areas , and the ratio of retinal neovascularization in a dose-dependent manner (Figure 5a,b). Furthermore, SPN significantly alleviated the neovascularization, vessel tortuosity, and dilated vessels of the OIR rats (Figure 6).

**Figure 7:**
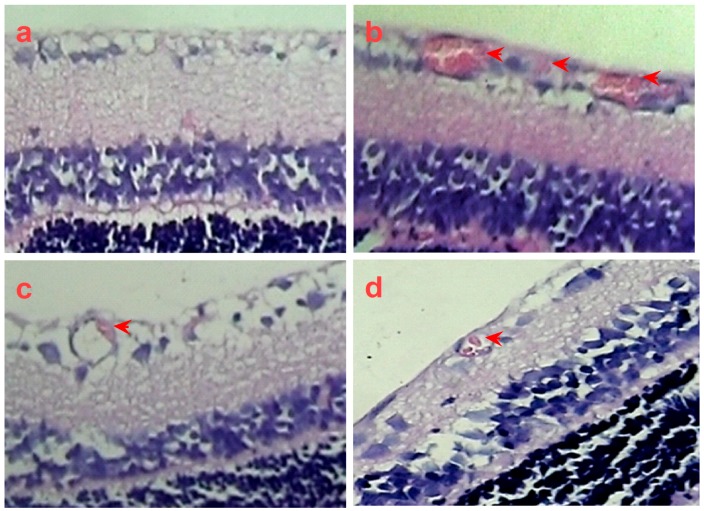
The in vivo effect of SPN on retinal neovascularization in H&E-stained retinal tissue sections (400×). Control group (**a**), rat pups fostered under normoxia condition; oxygen-induced retinopathy (OIR) rats were intraperitoneally administered with normal saline (negative control, **b**), 1.0 mg/kg Conbercept (positive control, **c**), or 10.0 mg/kg SPN (**d**). The arrows show neovascular nuclei or vessels. Ten rats were used for each group.

To further confirm the inhibitory effect of SPN on retinal neovascularization, vascular cell nuclei that extended beyond the internal limiting membrane were counted on H&E-stained retinal tissue sections. Nuclei anterior to the internal limiting membrane were not found among the retinal tissue sections in the control group (Figure 7a), but numerous neovascular nuclei or vessels were found in the eyes of OIR rat (Figure 7b), whereas the neovascular nuclei or vessels were remarkably reduced in the Conbercept (CBC) or SPN- treated groups (Figure 7c,d).

## 3. Discussion

**Figure 8:**
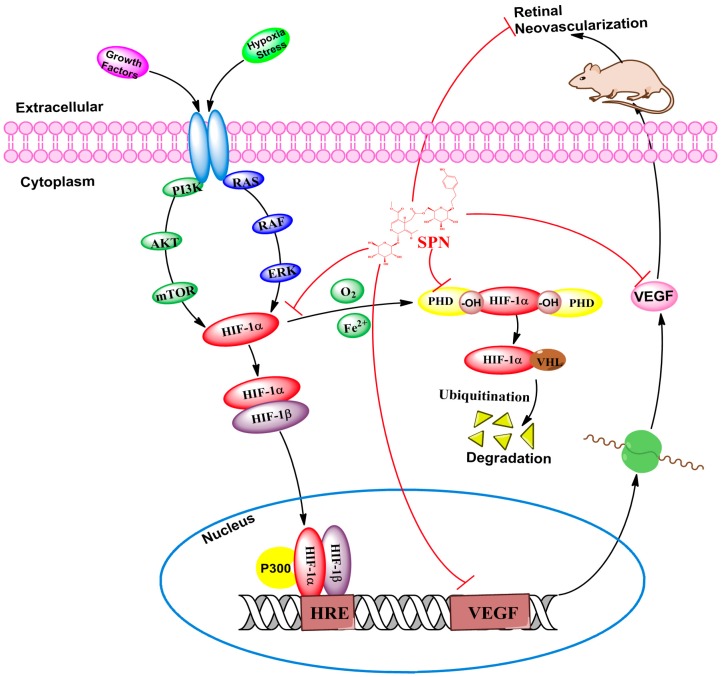
Effect of SPN on hypoxia-induced retinal angiogenesis.

The present study aimed to investigate whether SPN can prevent hypoxia-induced retinal neovascularization via inhibition of HIF-1α/VEGF signaling pathway in a VEGFA secretion model of ARPE-19 cells induced by CoCl_2_ in vitro and a neovascularization rat model of oxygen-induced retinopathy in vivo. Our results indicate that SPN at non-toxic concentrations (0.2–5.0 μg/mL) can indeed inhibit VEGFA secretion in ARPE-19 cells under hypoxia, and down-regulate the mRNA expressions of VEGFA and PHD-2 and the protein expressions of VEGFA, HIF-1α and PHD-2 in vitro. Furthermore, the results also show that SPN can prevent hypoxia-induced retinal neovascularization in vivo. The results suggest that SPN can alleviate hypoxia-induced retinal angiogenesis through suppression of the HIF-1α/VEGF signaling pathway (Figure 8). Therefore, our findings suggest that SPN may be effective against DR.

VEGFA plays the key role in the retinal neovascularization in the pathological process of DR, which involves in several functions, such as angiogenesis, vasculogenesis, endothelial cell growth and so on. RPE cells, play an important role in maintaining retinal homeostasis [36]. In the present study, we used CoCl_2_ to mimic hypoxia in cultured ARPE-19 cells to study the regulation of VEGFA expression. Our data revealed that exposure of 150 µM CoCl_2_ to the cells for 48 h increased the levels of VEGFA secretion, while 24 h exposure increased mRNA expression significantly in a dose dependent manner. The different effects with CoCl_2_ exposure may result in different times for mRNA transcription, translation and secretion of VEGFA. Furthermore, our data also demonstrate that SPN can simultaneously inhibit the mRNA transcription and protein expression of VEGFA.

RPE has important functions in both normal and pathological conditions in eyes. Growing evidence shows that RPE is associated with the pathogenesis of choroidal neovascularization (CNV), just as age-related macular degeneration (AMD). In healthy condition, RPE has a positive survival effect in the maintenance of the highly vascularized, permeable fenestrated choriocapillaris on its outer basal aspect, whereas the photoreceptor layer internal to it is completely avascular [40]. However, emerging evidence suggests that barrier leakage not only affects the inner blood–retina barriers (BRB) but also the outer BRB in early stage of DR. The outer BRB is comprised of tight junctions between RPE cells. The RPE is a multi-functional cell monolayer and is critically important to retinal health; it also is the biggest provider of VEGF in the eyes [41]. Our results suggest that inhibition of VEGFA in RPE by SPN is an important mechanism associated with its anti-DR effect.

HIF is a heterodimeric transcriptional factor that is activated and stabilized under hypoxic condition and it promotes the expressions of gene products [42], including angiogenesis factors (e.g., VEGF, TGF-β_3_) [43], erythropoiesis factor (e.g., EPO) [44], cell survival and proliferation factors (e.g., IGF-2, ID2, NOS) and so on [45,46]. HIF-1 is composed of two subunits of HIF-1α and HIF-1β. HIF-1α is an oxygen sensitive subunit and its expression is induced under hypoxic condition. In contrast, HIF-1β is constitutively expressed. HIF-1β is also known as aryl hydrocarbon nuclear translocator (ARNT), because it needs to binds with HIF-1α and AhR facilitating its translocation to the nucleus [39]. The expression of PHD-2 is up-regulated under hypoxia but the activity of PHD-2 is inhibited under low oxygen tension [35,36]. This counter action could explain that the expression of PHD-2 is increased by CoCl_2_, while it is down-regulated by SPN through inhibition of HIF-1α protein expression as previous reported [47]. In the present study, we found the mRNA expressions of VEGFA and PHD-2 and the protein expressions of VEGFA, HIF-1α and PHD-2 were induced in hypoxia-mimicking condition by CoCl_2_. Meanwhile, SPN could inhibit the increased expressions induced by CoCl_2_ in the dose-dependent manner through inhibition of HIF-1α/VEGF signaling pathway. In order to demonstrate the inhibitory effect of SPN on retinal neovascularization in vivo, the rat model of OIR was used to investigate the pharmacological effect of SPN on ischemic retinopathy induced by hyperoxia and followed by return to normoxia as previously reported [37]. Increased retinal neovascularization was found in the OIR rats and SPN alleviated the neovascularization, vessel tortuosity and dilated vessels significantly, suggesting SPN may be a potent inhibitor of retinal neovascularization via suppression of HIF-1α/VEGF signaling pathway.

Actually, HIF-1α undergoes quick degradation under normoxic conditions and normally has a very short half-life (about 5 min) [48]. In contrast, in hypoxic condition, several pathways have been shown to control HIF-1α stability and transcriptional activity via post-transnational modifications involving hydroxylation, acetylation, ubiquitination, and phosphorylation reactions, including hypoxic regulation pathway (pVHL-dependent or pVHL-independent) [49], growth factor signaling pathway [50], Mdm2 pathway [51], HSP90 pathway and so on [52]. Therefore, our further investigation into the molecular mechanisms associated with the effects of anti-retinal neovascularization by SPN should include the study of upstream regulation, degradation of HIF-1α and HRE transcription.

## 4. Materials and Methods 

### 4.1. Plant Material

The fruits of *Ligustrum lucidum* Ait. (*L. lucidum*) were purchased from a local market in Chengdu, Sichuan Province, China, and were identified by Prof. Xianming Lu of Chengdu University of Traditional Chinese Medicine (Chengdu, Sichuan, China). The voucher specimen (ZY0017) was deposited in the KangHong Pharmaceutical Group (Chengdu, Sichuan, China).

### 4.2. Reagents and Antibodies

Conbercept Ophthalmic Injection (a recombinant fusion protein and used for the treatment of retinal or choroidal neovascularization, Lot: 20110610B) was provided by Chengdu KangHong Pharmaceutical Group Co., Ltd. (Chengdu, Sichuan, China). Human VEGF Quantikine ELISA kits (Lot: DVE00) were provided by R&D Systems Inc. (Stillwater, MN, USA). DMEM/F12 medium, fetal bovine serum (FBS) and trypsin-EDTA solution were purchased from GIBCO Invitrogen (Carlsbad, CA, USA). Tetramethylethylenediamine (TEMED), DMSO, adenosine diphosphate (ADP), and ammonium sulfide were purchased from Sigma (St. Louis, MO, USA). 3-(5′-hydroxymethyl-2′-furyl)-1-benzylindazole (YC-1, a potent HIF-1α inhibitor), anti-HIF-1α antibody and anti-PHD-2 antibody were obtained from Abcam (Cambridge, MA, USA). PrimeScript RT reagent kit with gDNA Eraser and SYBR Premix Ex Taq™ were purchased from Takara-Bio (Kusatsu, Shiga, Japan).

### 4.3. Cell Culture

ARPE-19 cell line (Lot: 60279299) was purchased from the American Type Culture Collection (ATCC, Manassas, VA, USA), and the cells were cultured in DMEM/F12 medium with 10% FBS, 100 units/mL of penicillin and 100 μg/mL of streptomycin in an incubator at 37 °C with 5% CO_2_. After 90% confluence, the cells were digested by 0.25% trypsin–0.02% EDTA for the passages.

### 4.4. Extraction and Isolation of SPN

The air-dried fruits of *L. lucidum* (1.0 kg) were ground, and reflux-extracted with 70% ethanol for three times (8000 mL, 1 h each). After filtration and evaporation, the ethanol extract was yielded (136.7 g). The ethanol extract (5.0 g) was dissolved in methanol and subjected to column chromatography on an AKTA system (AKTA Basic, GE Healthcare, Pittsburgh, PA, USA) using a Sepax Gp-C18 column (250 mm × 10 mm, 5 μm) and eluted at 10 mL/min flow rate and wavelength 254 nm using 1% acetic acid (solvent A) and MeOH (solvent B) with the following gradient of composition: starting with 5% solvent B for 10 min and changing to 20% during the next 10 min, followed by a second ramp to 100% B in 40 min, maintained for 10 min, and then was purified by preparative HPLC on an AKTA Basic system (GE Healthcare, Pittsburgh, PA, USA) to yield the SPN (28.3 mg, purity 98.3%, RT 17.2 min). The chemical structure of SPN was confirmed by MS and NMR as described in a previous report [53].

### 4.5. Determination of VEGFA Secretion by ARPE-19 Cells under Chemical Induced Hypoxia

According to previous report from the literature [27], ARPE-19 cells were seeded into 96-well plates at a density of 8 × 10^3^ cells per well. After being cultured for 24 h, the culture medium was replaced with fresh FBS free DMEM/F12 medium. Then, the cells were treated with, different concentrations (0.2, 1.0 and 5.0 μg/mL) of SPN, YC-1 (1.0 μg/mL) as positive control, or the culture medium containing same DMSO concentration of the SPN solution as control. 150 μM CoCl_2_ was added to mimic hypoxic condition 24 h later. Conditioned medium was collected 48 h later and centrifuged at 800× *g* for 5 min, and the supernatants were transferred to vials and stored at −80 °C for further analysis. All experiments were performed at least three times in triplicate. The concentrations of VEGFA protein in the conditioned media were measured using the human VEGF ELISA kits according to the manufacturer’s instruction. Absorbance values (450 nm) were recorded in triplicate using M_5_ Microplate Reader (SpectraMax M_5_, Molecular Devices, Sunnyvale, CA, USA). The concentrations of VEGFA were calculated from a standard curve. The sensitivity of the VEGFA kit was 5.0 pg/mL.

### 4.6. RNA Isolation and Analysis of the mRNA Expressions of VEGFA, HIF-1α and PHD-2

ARPE-19 cells were plated at a density of 1×10^5^ cells/well in 6-well plate and allowed to adhere overnight, then submitted to the same treatments as described above. After the cells being cultured for 24 h, the culture medium was withdrawn, and the cells were washed with cold PBS before harvest. The cell pellets were collected for mRNA extraction after microcentrifuging at 800× *g* for 5 min at 4 °C. Total RNA from ARPE-19 cells was isolated with TRIzol reagent. cDNA was synthesized with a cDNA synthesis kit according to the manufacturer’s protocol. The relative levels of each gene mRNA transcripts to β-actin were determined by qRT-PCR using the SYBR pre-mixed system and specific primers. The primer sequences for VEGFA, HIF-1α, PHD-2 and β-actin were as follows: Homo VEGFA 5′-CGA AAC CAT GAA CTT TCT GC-3′ (forward) and 5′-CCT CAG TGG GCA CAC ACT CC-3′ (reverse). Homo HIF-1α 5′-ACA AGT CAC CAC AGG ACA G-3′ (forward) and 5′-AGG GAG AAA ATC AAG TCG-3′ (reverse). Homo PHD-2 5′-AAA CCA TTG GGC TGC TCA T-3′ (forward) and 5′-CGT ACA TAA CCC GTT CCA TTG-3′ (reverse). Homo β-actin 5′-AGC GGG AAA TCG TGC GTG AC-3′ (forward) and 5′-AGT TTC GTG GAT GCC ACA GGA C-3′ (reverse). qRT-PCR was performed by Opticon 3 continuous fluorescence detector (MJ Research Inc., Waltham, MA, USA). The comparative cycle of threshold fluorescence (Ct) method was used and the relative transcript amount of the target gene was normalized to that of β-actin using the 2^−ΔΔCt^ method.

### 4.7. Western Blots Analysis of HIF-1α and PHD-2 Proteins

ARPE-19 cells were seeded at a density of 2.0 × 10^5^ cells/bottle in culture bottles and submitted to the same treatments as described above. After being cultured for 24 h, the culture medium was withdrawn and the cells were washed with cold PBS for harvest. The cell pellets were disrupted in cell RIPA buffer (0.5% NP-40, 50 mM Tris-HCl, 120 mM NaCl, 1 mM EDTA, 0.1 mM Na_3_VO_4_, 1 mM NaF, 1 mM PMSF, and 1 μg/mL leupeptin, pH 7.5), then the lysates were centrifuged at 12,000 rpm for 15 min at 4 °C. The protein concentrations were determined using the BCA method, after which equal amount of protein (30 μg) was electrophoresed on 7.5% density of SDS-acrylamide gels. Following electrophoresis, the proteins were transferred from the gel to a nitrocellulose membrane using an electric transfer system. Non-specific binding was blocked with 5% skim milk in TBST buffer (5 mM Tris-HCl, 136 mM NaCl and 0.1% Tween-20, pH 7.6) for 1 h. The blots were incubated with antibodies against HIF-1α (1:2000), PHD-2 (1:1500) or β-actin (1:800) overnight at 4 °C and were washed three times with 1× TBST. Then, the blots were incubated for 1 h at room temperature with a 1:4000 dilution of horseradish peroxidase-labeled anti-rabbit or anti-mouse IgG and washed three times with 1× TBST, the membranes were developed by incubation within the ECL Western detection reagents.

### 4.8. Rat Model of Oxygen-Induced Retinopathy (OIR)

All animal experiments were performed strictly in accordance with University’s guidelines and were approved by the Committee on Use and Care of Animals of Sichuan University (Permit No. 20130146, Chengdu, Sichuan, China). Ten pregnant (16-20-week old and body weight 300–350 g, for OIR model study) Sprague Dawley (SD) rats (SPF Grade, Certificate No. SCXK2013-24) were purchased from Experimental Animal Centre, Sichuan Provincial Academy of Medical Sciences in China (Chengdu, Sichuan, China). OIR was induced in SD rat pups according to a protocol as previously described [39]. In brief, fifty SD rat pups with their nursing mothers were placed in an 80% oxygen atmosphere on PD 7 for 5 days. After being returned to normoxia conditions on PD 12, the rat pups were intraperitoneally (i.p.) administered with 0.5 mL of Conbercept ophthalmic injection (1.0 mg/kg), different doses of SPN (5.0 and 10.0 mg/kg) or normal saline (NS). Five days later (on PD 17), the rat pups were sacrificed (the pups in the control group were bred under normoxia condition until PD 17), and the eyes were enucleated and fixed in fresh 4% paraformaldehyde for 2 h. Eyecups were dissected and the retinal flat mounts were created and stained with ADPase stain as described previously [54]. The retinal avascular areas were assessed through visualization of the retinal vasculature using infusion of ADPase staining examined under fluorescent microscopy. In each whole mount, the total areas of pre-retinal neovascular area were measured using Image-pro Plus System and expressed as the percentage of the respective average in relation to total retinal areas. The retinas were also histologically examined. Serial sections of paraffin-embedded small pieces of retina (6 mm) were stained with hematoxylin & eosin (H&E). Images were taken under microscopy, and endothelial nuclei that extended beyond the inner limiting membrane into the vitreous were manually counted in a blind manner [55].

### 4.9. Statistical Analysis

All the data were reported as mean ± standard deviation (SD). Statistical significance of the data were analyzed by one-way univariate analysis of variance (ANOVA) for comparing data from more than two groups. A difference at *p* < 0.05 was considered to be statistically significant (as marked as *). The higher significance level was set at *p* < 0.01 (as marked as **).

## 5. Conclusions

In the present study, we demonstrated that SPN plays an important role in inhibition of VEGFA secretion through down-regulating mRNA expressions of VEGFA and PHD-2, and protein expressions of VEGFA, HIF-1α and PHD-2 in cultured ARPE-19 cells in vitro. Furthermore, we also demonstrate the inhibitory effect of SPN on retinal neovascularization in a rat model of OIR in vivo. These results may provide important insights into potential discovery and development of SPN as a novel agent for the treatment of DR clinically. However, more studies including other mouse retinal damage model induced by the oxidative damage of the blue-light irradiation and extramitochondrial metabolism in the rod outer segments [56], are needed to further investigate the pharmacological and toxic effects of SPN on additional animal models and associated mechanism(s) against DR.
